# Blood-Based Epigenetic Age Acceleration and Incident Colorectal Cancer Risk: Findings from a Population-Based Case–Control Study

**DOI:** 10.3390/ijms25094850

**Published:** 2024-04-29

**Authors:** Sofia Malyutina, Olga Chervova, Vladimir Maximov, Tatiana Nikitenko, Andrew Ryabikov, Mikhail Voevoda

**Affiliations:** 1 Research Institute of Internal and Preventive Medicine-Branch of Institute of Cytology and Genetics SB RAS, Novosibirsk 630089, Russia; medik11@mail.ru (V.M.); t_nikitenko_72@mail.ru (T.N.); a_ryabikov@hotmail.com (A.R.); mvoevoda@ya.ru (M.V.); 2 University College London, London WC1E6BT, UK; o.chervova@ucl.ac.uk

**Keywords:** DNA methylation, epigenetic age, ageing, colorectal cancer, case–control, population, HAPIEE project

## Abstract

This study investigates the association between epigenetic age acceleration (EAA) derived from DNA methylation and the risk of incident colorectal cancer (CRC). We utilized data from a random population sample of 9,360 individuals (men and women, aged 45–69) from the HAPIEE Study who had been followed up for 16 years. A nested case–control design yielded 35 incident CRC cases and 354 matched controls. Six baseline epigenetic age (EA) measures (Horvath, Hannum, PhenoAge, Skin and Blood (SB), BLUP, and Elastic Net (EN)) were calculated along with their respective EAAs. After adjustment, the odds ratios (ORs) for CRC risk per decile increase in EAA ranged from 1.20 (95% CI: 1.04–1.39) to 1.44 (95% CI: 1.21–1.76) for the Horvath, Hannum, PhenoAge, and BLUP measures. Conversely, the SB and EN EAA measures showed borderline inverse associations with ORs of 0.86–0.87 (95% CI: 0.76–0.99). Tertile analysis reinforced a positive association between CRC risk and four EAA measures (Horvath, Hannum, PhenoAge, and BLUP) and a modest inverse relationship with EN EAA. Our findings from a prospective population-based-case-control study indicate a direct association between incident CRC and four markers of accelerated baseline epigenetic age. In contrast, two markers showed a negative association or no association. These results warrant further exploration in larger cohorts and may have implications for CRC risk assessment and prevention.

## 1. Introduction

A global trend of increasing of life expectancy is leading to ageing of the world’s population and the accumulation of age-dependent diseases. According to the United Nation’s estimates, the world’s population is expected to reach 8.6 billion people by 2030, with more than 1.4 billion people over the age of 60 [1].

Cancer occupies the second position among the top ten causes of death in the elderly aged 65+, according to CDC data from 2021 [2]. Colorectal cancer (CRC) is the second most common cancer type in women and the third most common in men, accounting for about 10% of all cancers globally [3,4]. The mortality rate of CRC is estimated as 7.2 and 11.0 per 100,000 in women and men, respectively [5]; the incidence rate of CRC is 14.3 per 100,000 in women and 20.6 per 100,000 in men, and the WHO estimates an increase of about 80% in new cases and deaths from CRC by 2030 [4]. According to the recent estimates based on the GLOBOCAN data, the burden of CRC is projected to increase to 3.2 million new cases and 1.6 million deaths by 2040 [6].

CRC rarely occurs at a young age; its incidence increases after the age of 50 years, reaching a maximum by age 75–80 years. More than 90% of CRC cases occur in people over 50 years of age [4,7]. Small proportions of CRC are due to inherited forms with known mechanisms (e.g., familial adenomatous polyposis, FAP; and Lynch syndrome) or with an associated hereditary component that has not been well established (nearly 20%); however, the majority of CRC cases are sporadic [4].

From a genetic side, CRC development involves the accumulation of mutations leading to oncogene activation and suppressor-gene inactivation; a second developmental route involves the accumulation of mutations leading to defects in DNA repair [8,9]. For instance, FAP is attributed to loss-of-function mutations in the adenomatous polypus coli (APC) tumour suppressor gene; mutations in genes for p53 and K-RAS and other oncoproteins affect the proliferation of precancerous cells and increase the potential to develop adenocarcinoma [10]. In a strategy of cancer chemoprevention, the recent review by Ramesh et al. describes novel compounds (sulindac derivates) that suppress the growth of CRC cells by inhibiting cGMP phosphodiesterases and suppressing Wnt/β-catenin transcription [10].

According to the multifactorial nature of CRC, in addition to genetic and epigenetic alterations and hereditary risks, multiple risk factors have impacts on CRC development, including older age, male sex, ethnicity, inflammatory bowel disease, diabetes, obesity, behavioural and environmental factors such as smoking, certain patterns of diet, alcohol intake, low physical activity, and early life exposures [11,12,13,14,15].

Ageing is one of the most important risk factors for CRC [16]; its contribution largely depends on the ‘biological age’ that reflects an individual’s rate of health decline. It is worth mentioning that harmful environmental and behaviour factors themselves might affect the biological ageing processes leading to cellular vulnerability, cell senescence, genomic and epigenomic instability, mitochondrial dysfunction, and telomere attrition [17].

Epigenetic modifications such as DNA methylation (DNAm) have been shown to be the most accurate molecular readouts of ageing but their possible functional roles remain poorly understood [18,19,20]. It has been shown that DNAm levels at specific CpG sites in the genome may predict chronological age [21,22,23]; consequently, the concept of ‘epigenetic clocks’ has been developed. To date, over 30 DNAm-based estimators of ‘epigenetic age’ have been constructed, including the “first generation” blood-based (Hannum, 2013) [24] and pan-tissue-based clocks (Horvath, 2013) [25] used to predict chronological age; the “second generation” of tools captured age-related and functional-phenotype modifications [26,27,28,29,30], and later development yielded cancer-specific epigenetic clocks that approximate a mitotic clock in normal and cancer tissues [31,32,33].

A number of studies have shown associations between the acceleration of epigenetic age (EAA) and risk of mortality, as summarized in various meta-analyses [34,35,36,37,38]. The evaluation of relationship between EAA and CRC have reported mostly positive associations, but negative relations and equivocal results have also been reported [20,39,40,41,42,43,44,45]. The inconsistency in the findings is related to the type of CRC-related outcome used, the use of various calculators of EA and EAA, blood or tissue analysis of DNAm, study design and sampling, sex and ethnic heterogeneity, etc. In this context, further investigations of the relationship between epigenetic age and CRC is relevant for a spectrum of end points (incidence, prognosis, and mortality), in the design of prospective studies with large sample numbers and different populations.

Basically, we used the Russian population cohort of the HAPIEE Study (Novosibirsk), which was established in 2003–2005 and followed up longitudinally, to investigate the association between biomarkers of ageing (DNAm, leukocyte telomere length, and mitochondrial DNA copy number) and risks for a number of age-related outcomes. The findings obtained previously are reported elsewhere [46,47,48,49,50,51].

In the present paper, we aimed to investigate the relationship between six markers of epigenetic age acceleration estimated from blood DNA methylation and incident colorectal cancer during a 16-year follow-up in a nested case–control study from a population cohort.

## 2. Results

### 2.1. General Baseline Characteristics of the Studied CRC and Control Groups

We examined a random population sample (n = 9360, men/women, 45–69, from the HAPIEE Study) in 2003/2005 (baseline) and followed up participants for 16 years. Using a nested case–control study design, we selected participants with incident colorectal cancer (CRC) among those free from baseline cancer and selected a sex- and age-stratified controls. After selecting a limited subset for DNAm profiling and subsequent quality control procedures, the analytical sample comprised 35 cases and 354 controls. The baseline characteristics of the case and control groups are presented in Table 1.

The incident CRC cases and controls were well-matched for age, sex, and most baseline characteristics, with the exception of higher high-density lipoprotein cholesterol (HDLC) levels observed among CRC cases compared to controls.

The mean age (SD; median) of CRC diagnosis was 68.4 years (8.96; 69.21), and the mean time between blood draw and registration of cancer was 8.29 years (1.85; 15.26). The mean age of CRC registration and mean time to event in the case group were close to those in the entire cohort sample of incident CRC (68.5, SD = 8.00 and 7.74, SD = 4.53 years).

**Figure 1:**
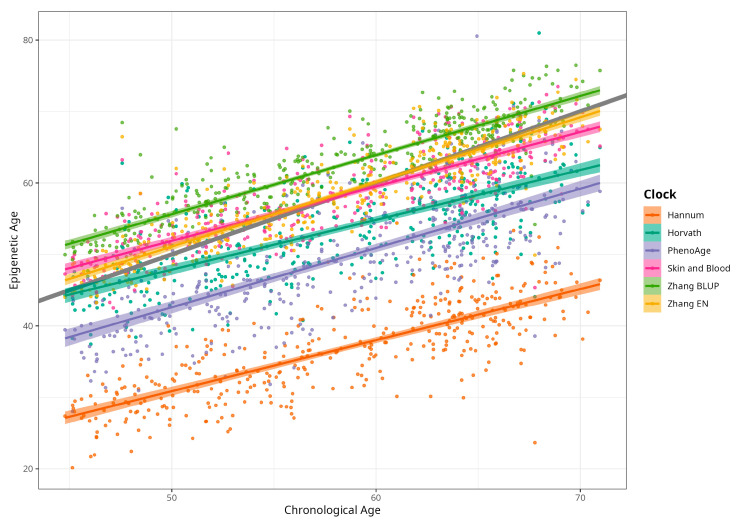
Scatterplots of chronological vs. epigenetic age by Horvath, Hannum, PhenoAge, Skin and Blood, BLUP, and Elastic Net clocks. Diagonal grey line corresponds to the Predicted age equal to the chronological age, and coloured lines correspond to the linear regression result (n = 389, men and women, CRC cases and control).

**Figure 2:**
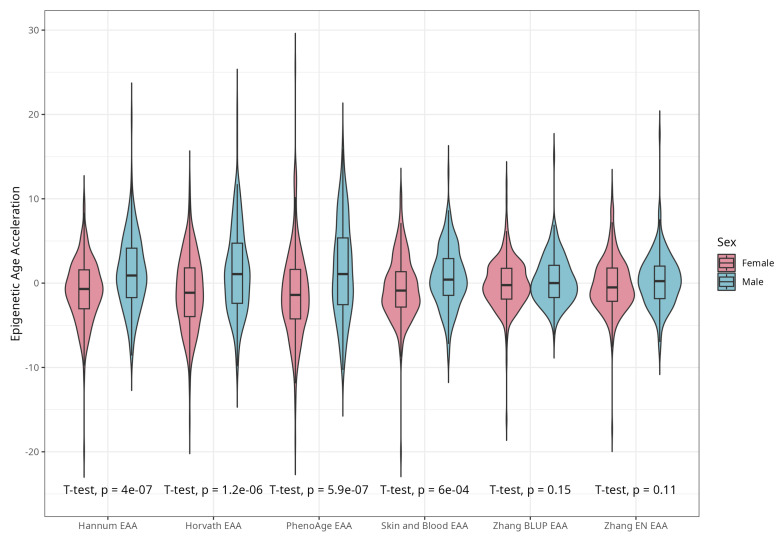
Violin plots of EAA stratified by sex for Horvath, Hannum, PhenoAge, Skin and Blood, BLUP, and Elastic Net clocks (residuals from regression of EA by CA; no. = 389, men and women, CRC cases and controls).

DNAm ages calculated by Horvath’s, Skin and Blood, BLUP and EN clocks were close to participants’ CA; the corresponding median differences were −5.02, −0.06, 4.14, and 0.13 years. The corresponding mean (SD) values were −4.66 (5.25), −0.07 (4.08), 4.19 (3.16), and 0.30 (3.29) years for difference between Horvath’s, Skin and Blood, BLUP, and EN epigenetic ages and CA, respectively. As expected, Hannum and PhenoAge EAs were less close to CA, with median differences −21.60 and −9.11 years, respectively, and mean differences (SDs) of −21.51 (4.58) and −8.83 (5.78) years. Scatterplots of chronological vs. epigenetic age for six markers are presented in Figure 1. The correlation coefficients between CA and EA were significant and ranged from 0.721 (Horvath) to 0.898 (BLUP), with *p* < 0.001 for all six measures (Supplementary Material, Figure S1). Violin plots of sex-specific epigenetic age acceleration (EAA) calculated as the residuals from regressing epigenetic age on chronological age (referred to furthermore as EAAs) for six clocks are shown in Figure 2. The mean EAAs, as expected, were higher in men compared to women for the majority of markers (*p*-value < 6 × 10^−4^), except for BLUP and EN.

**Figure 3:**
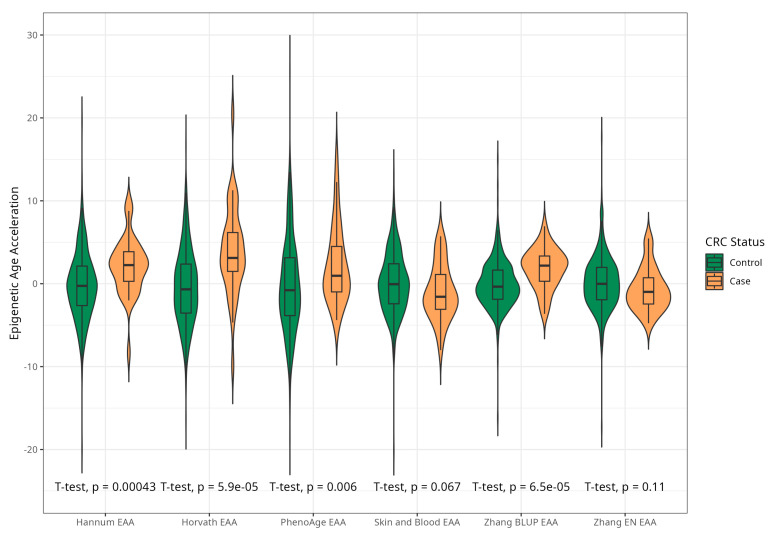
Violin plots of EAA in CRC cases and controls for Horvath, Hannum, PhenoAge, Skin and Blood, BLUP, and Elastic Net clocks (residuals from regression of EA by CA; no. = 389, men and women, CRC cases and controls).

**Figure 4:**
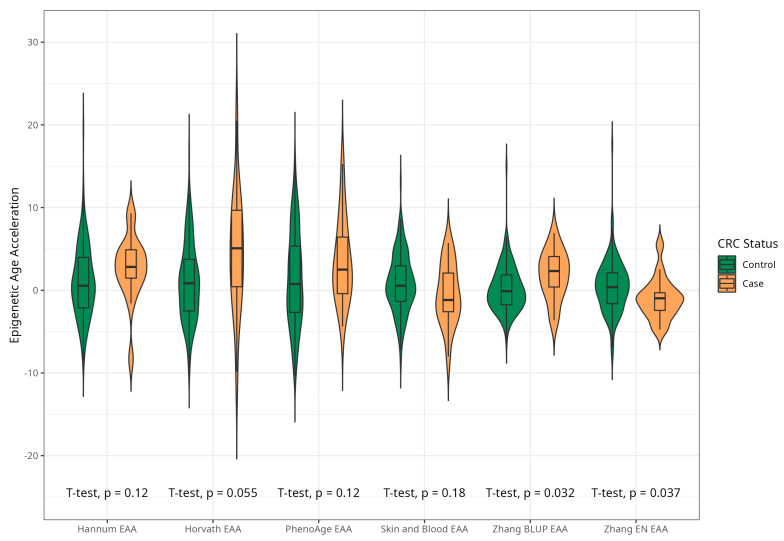
Violin plots of EAA in CRC cases and controls for Horvath, Hannum, Levine, Skin and Blood, BLUP, and Elastic Net clocks in men (residuals from regression of EA by CA; no. = 168, men, CRC cases and controls).

**Figure 5:**
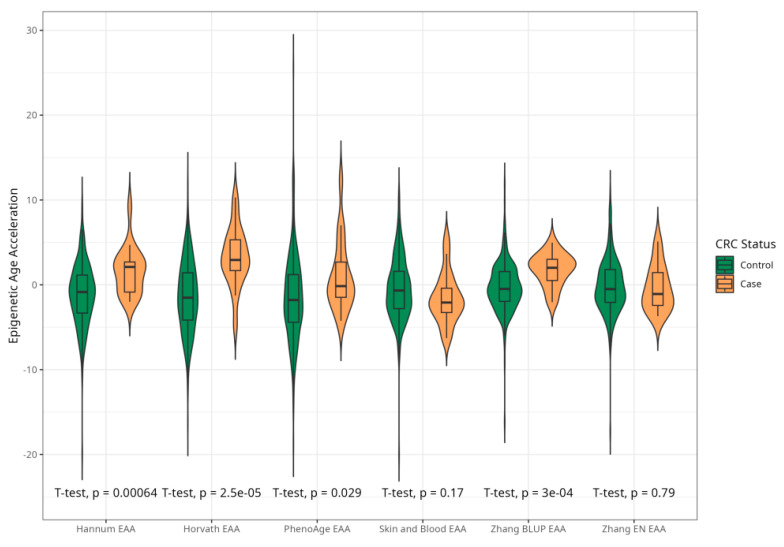
Violin plots of differences in EAA between CRC cases and controls for Horvath, Hannum, PhenoAge, Skin and Blood, BLUPs and Elastic Net clocks in women (residuals from regression of EA by CA; no. = 221, women, CRC cases and controls).

The mean EAAs for four markers were significantly higher in CRC cases compared to the control group, with 3.91 (5.45) vs. −0.43 (4.53), *p* < 0.001 for Horvath; 2.15 (3.41) vs. −0.24 (4.10), *p* < 0.001 for Hannum; 2.29 (4.73) vs. −0.22 (5.69), *p* = 0.005 for PhenoAge; and 1.83 (2.41) vs. −0.11(2.90) *p* < 0.001 for BLUP. The values of EAAs from Skin and Blood and EN clocks were similar in cases and controls (Table 1; Figure 3). These relationships were consistent among women. Among men, only BLUP, EN, and Horvath EAAs were higher in CRC cases vs. controls (Figure 4 and Figure 5).

### 2.2. Association between Baseline Epigenetic Age Acceleration by Six Markers and Risk of CRC

We assessed the odds ratios (ORs) of incident CRC per decile increment of EAAs as a continuous variable in multivariable-adjusted logistic regression for six epigenetic clocks. The results are presented in Table 2.

The ORs of CRC risk per decile increase of EAA were 1.43 (95% CI 1.22–1.67) for Horvath; 1.29 (95% CI 1.11–1.48) for Hannum; 1.20 (95% CI 1.05–1.37) for Levine; and 1.34 (95% CI 1.16–1.55) for BLUP clocks. The ORs were less than 1.0 for SB and EN clocks 0.88 (95% CI 0.77–0.99) and 0.90 (95% CI 0.78–1.02), respectively, in age-adjusted models. In fully adjusted Model 4, the associations remained positive and significant for epigenetic age acceleration from Horvath, Hannum, PhenoAge, and BLUP clocks with ORs between 1.44 (95% CI 1.21–1.76) and 1.20 (95% CI 1.04–1.39) per decile increase in EAA regardless of age, sex, smoking, SBP, TC or HDLC, BMI or WHR, FPG, alcohol intake, or education. ORs for CRC showed modest inverse relationships for Skin and Blood and EN EAAs: 0.86–0.87 (95% CI 0.76–0.99).

The estimates of associations split by sex are presented in the Supplementary Materials, Table S1. The relationships between EAA and CRC in men and women were in the same direction compared to the pooled results. In the multivariable-adjusted models, the positive associations remained significant for EAAs for Horvath and Hannum clocks in women, and for the BLUP clock in both sexes; inverse relationships remained significant for Skin and Blood and EN clocks in men.

Odds ratios for incident CRC by tertiles of EAA measures are presented in Table 3. The bottom tertile was used as a reference in a logistic regression model applying the same covariates. Tertile analyses supported positive associations between CRC incidence and four EAA measures (from Horvath, Hannum, PhenoAge, and BLUP clocks) and an inverse relationship of CRC incidence with EN EAA.

### 2.3. Sensitivity Analyses: Associations between Baseline Epigenetic Age Acceleration by Six Markers and Risk of CRC Excluding Early Cases and Against Extended Control

To ensure the robustness of our findings, we conducted a sensitivity analysis excluding incident CRC cases that occurred within the first three years after the baseline examination. We re-estimated the associations between the six measures EAA and incident CRC using this restricted sample. The results were highly consistent with the coefficients obtained in the primary set of CRC cases (Supplementary Material, Table S2).

In a further sensitivity analysis, we expanded the control group to include all participants with available DNA methylation measurements, regardless of their incident cancer status (n = 424 in total). We then re-evaluated the associations between the six measures of baseline epigenetic age acceleration (EAA) and colorectal cancer risk using this expanded dataset (Supplementary Material, Table S3). The odds ratios of CRC risk per decile increase in baseline EAA were similar to those obtained in the primary nested case–control analysis for five of the six EAA markers. However, the association for EN EAA was attenuated and no longer statistically significant in this expanded analysis. We also conducted stratified analyses in the expanded dataset, estimating the EAA–CRC associations separately for men and women and by tertiles of EAA (data not shown). The results from these stratified analyses did not substantially differ from those obtained in the primary analysis.

## 3. Discussion

### 3.1. The Relationship between Measures of Epigenetic Age Acceleration and CRC

In our study, which was designed as a nested case–control study, we selected a group of incident CRC cases that occurred during a 16-year follow-up and selected age- and sex-frequency-matched controls from a population-based cohort (Novosibirsk, the HAPIEE Study). Epigenetic age was derived from DNAm data for six clocks. In our dataset, EA by Horvath’s, Skin and Blood, BLUP, and EN clocks were similar to participants’ chronological ages; predictions by Hannum’s and Levine’s clocks were less close to CA.

The positive deviation of EA from chronological age is considered ‘accelerated biological aging’. In our sample, incident CRC was positively associated with four EAA markers with adjusted ORs that ranged from 1.20 to 1.44 per decile increase in EAA (for Horvath, Hannum, PhenoAge, BLUP clocks). The relationships between the risk of CRC and EAA by Skin and Blood and EN clocks were weakly inverse, with adjusted ORs of 0.86–0.87 and borderline significance. When EAA was classified into tertiles, the analysis supported strong positive associations between incident CRC and four EAA measures (for Horvath, Hannum, PhenoAge, and BLUP clocks) and a modest negative relationship of CRC with EN EAA.

Our findings of positive associations between a number of EAA measures and risk of incident CRC are consistent with the EPIC-Italy project [39], and partly consistent with a series of analyses in the Melbourne Collaborative Cohort Study (MCCS) [40,41]. Also, our results are in line with the recent analysis of DNAm data from CRC-related tissue samples by Widayati et al. [42], and with a case–control study of CRC survival in the German DACHS Study [44].

For example, the EPIC-Italy project (n = 845; 10-year follow-up; 235 breast cancers, 166 CRC; and five blood DNAm clocks) showed significant acceleration of epigenetic age in incident CRC cases among men compared to their CRC free counterparts for Horvath’s and FHL’s clocks, and a similar but weaker trend for Hannum’s clock. Specifically, male participants with CRC were 1.6 and 2.5 years older (Horvath, Hannum) than CRC free men [39]. In early analyses from the Melbourne Collaborative Cohort Study using the first-generation epigenetic clocks (pooled seven case–control studies; no. = 3216 cancer cases, matched controls; and 1726 deaths), Dugue et al. reported in 2018 an association between EAA and increased risk of pooled cancers, ranging from 4% to 9% increased incidence per 5-year age acceleration of EA (Hannum and Horvath), and from 4% to 6% increased risk of cancer death (Hannum, EEAA Hannum). Specifically for CRC risk, the ORs ranged 1.05–1.10 per 5-year increase in EAA; the direction of reported estimates is consistent with our findings, while they were weaker and statistically not significant in MCCS dataset [40]. In more recent analyses in the MCCS, 2021, using second-generation epigenetic clocks, the authors observed strong associations between age acceleration and CRC incidence, with adjusted ORs of 1.22 and 1.19 per 1SD increase in EAA by PhenoAge and GrimAge clocks, respectively [41], which is close to our estimate for PhenoAge.

In the recent analyses by Widayati et al. in 2023, which were based on 14 open-access data sets from NCBI GEO and ArrayExpress, the authors investigated the DNAm data from 1845 tissue samples (CRC tumour, normal colon adjacent to tumour, colorectal adenoma, and normal colon from healthy persons) and studied their relationships with EA and EAA from 11 clocks. It was shown that EAA from Horvath, PhenoAge, Wu, EpiTOC, HypoClock, and MiAge clocks captured differences between every tissue except for between adenomas and healthy tissue. Most EAAs differed between tumour samples and normal or healthy samples. In results supportive of our findings, EAAs for tumour tissue were significantly higher than those for normal and healthy samples by PhenoAge, Skin and Blood, Wu, BLUP, EpiTOC, and MiAg clocks [42].

In the case–control study of CRC survival in the German DACHS Study (n = 2206 CRC patients; follow-up for 6.2 years; and 596 CRC deaths), the HRs of CRC death were 1.30, 1.15, and 1.26 per 1SD of EAA increase in DNAmMRscore, PhenoAge, and GrimAge models [44].

A recent Mendelian randomization (MR) study and meta-analysis [20] (n = 34,710; UK Biobank, FinnGen, and several cancer consortia) provided evidence of a causal relationship between genetically predicted GrimAge acceleration and CRC risk with OR = 1.12 per 1 year increase in EAA by GrimAge, which was independent of multiple corrections and consistent across cohorts and sexes. The authors did not find evidence for causality between other measures of EAA and CRC.

On the other hand, Wang et al. reported in 2020 on samples of normal colon and revealed, in contrast, the deceleration of EA in a high-risk group for CRC compared to a low-risk group (by PhenoAge) [43]. At the same time, after adding the CRC samples to the analysis, PhenoAge and EpiTOC clocks were significantly accelerated in the CRC samples. The authors did not observe associations between CRC risk and Horvath or GrimAge measures. Also, our findings are in contradiction with the Scottish Family Health Study [45], which did not observe an association between EA acceleration and incident bowel cancer.

The process of ageing is accompanied by epigenetic alterations, including changes in DNAm [17]. As well, alterations in DNAm patterns are a common form of epigenetic changes in CRC. They contribute to abnormal cell growth of the intestine, followed by adenoma development and progression to cancer [16]. Translational research has also identified DNAm-based epigenetic age acceleration among potential biomarkers involved in CRC cancerogenesis.

The deviation of EA from CA was shown to be associated with consensus molecular subtypes, a gene expression-based molecular classification established by the Colorectal Cancer Subtyping Consortium [52]. A recent GWAS has revealed 137 genetic loci associated with epigenetic age acceleration [53]. Using genetically predicted EA acceleration, a recent MR study provided evidence that genetically predicted GrimAge acceleration may increase the risk of CRC [20], which supports the suggestion of a causal effect.

Although little is known about its underlying mechanisms, EA acceleration may plausibly influence cancer risk via hormonal, inflammatory, and metabolic processes [20]. Additionally, age acceleration may capture the accumulation over a lifetime of exposures associated with both ageing and outcomes [40]. On the other hand, the previous demonstration of decelerating EA in normal colon tissue of subjects with CRC [43] suggests that cancerogenesis may involve disruption, rather than only acceleration of the epigenetic maintenance system; this may reflect expansion of a stem cell pool that further increases CRC risk.

There is heterogeneity in the findings about associations between CRC risk and EAA. In line with our observations, some studies have found that accelerated EA, measured by certain epigenetic clocks like GrimAge, may indicate increased CRC risk, while other clocks show no association or even an inverse association [4]. For example, the PhenoAge clock revealed epigenetic age deceleration in the normal colon mucosa of high-risk CRC individuals compared to that of low-risk individuals [43], suggesting that a dysfunctional epigenetic ageing process is occurring in those at elevated risk. The lack of significant case–control differences in our study for Skin and Blood and Zhang EN EAAs likely explains why the odds ratios for CRC risk trended in the opposite direction for these two clocks compared to the other four. The inconsistencies across different epigenetic clocks may be due to variations in their training data, CpG sites, clinical inputs, and statistical methods. Also, the inconsistency might be related to the heterogeneity of the studied CRC outcomes (such as incidence, progression, and survival); study design and sampling; and the age-, ethnic-, sex-composition, and population-specific characteristics of morbidity, risk-factor profiles, and environmental exposures. More research using open-access clinical data is needed to optimise clocks for robustly estimating CRC risk and prognosis [42].

### 3.2. Study Limitations and Strengths

Our findings should be considered while recognising their potential limitations. We have a moderate sample size (n = 389; 35 cases vs. 354 controls). This small number of CRC cases might limit the statistical power and could influence the generalizability of the findings to the population. However, our nested case–control analysis included a random age- and sex-stratified set of CRC cases selected from a complete sample of incident cases of colorectal cancer (n = 154) that developed in a large-scale cohort (9360) within a long-term follow-up of 16 years. We ensured the completeness of cancer registration by checking multiple sources of information for case ascertainment (including the Cancer Register, Mortality Register, and two repeated examinations of the cohort). The controls satisfied strict exclusion criteria and were frequency-matched to cases by age and sex. We therefore believe that this study’s design ensures that it is representative of the pattern of CRC occurrence for the studied population.

We observed higher EAAs in men compared to women for the majority of the markers studied. Taking into account a known difference in DNAm and in epigenetic age acceleration between sexes [33,54], as well as sexual dimorphism in CRC, the sex distribution in our study was uniform among cases and controls (nearly 50–60%) and we adjusted the estimates by sex. Also, the ORs in the sensitivity analyses split by sex were of similar values to the pooled results from a set of EAA measures associated significantly with CRC among both men and women.

We could not exclude the impact of other potential confounding factors, such as an unhealthy diet (low fruits and vegetables intake; red meat intake), socio-economic status (SES), etc. To mitigate this shortcoming, we tested models that included frequency of alcohol intake (5 categories), fruits and vegetables intake (by tertiles), which did not materially change the results. To account for SES, we used education level, which serves as a proxy for SES indicators. However, the contribution of diet or SES parameters merits specific separate analyses in further studies.

Another concern is that measures of DNAm methylation may reflect molecular changes due to cancerogenesis or treatment; consequently, early follow-up or retrospective design may capture reverse causation. To exclude this problem, we used a prospective design and included only incident CRC cases. Also, we fulfilled a sensitivity analysis excluding CRC cases within the first three years after the baseline blood draw that did not noticeably alter the results.

Finally, a limitation might arise from the use of a ‘super control’ group (without other incident cancers and alive during follow-up) that could inflate the ORs by being healthier than general population and less likely to be genetically prone to develop cancer [20]. To test this effect and the robustness of the estimates, we ran a secondary analysis using an extended control group (without exclusion of incident cancers other than CRC); this analysis demonstrated the associations close to initial dataset’s results.

Our study has also a number of strengths. To our view, this is the first prospective case–control study of the relationship between EAA and the risk of incident CRC in a Russian population sample or an eastern European population.

It is important to note that we used six measures of EAA (Horvath, Hannum, PhenoAge, Skin and Blood, BLUP and EN clocks) that were constructed as blood-based, pan-tissue or phenotype-based clocks, and we focused on various aspects of ageing, such as prediction of chronological age, or considered as a marker of age-related diseases and mortality.

Finally, our data provide the first evidence of an association between several measures of epigenetic age acceleration and incident colorectal cancer independent of other factors in a previously understudied population.

## 4. Materials and Methods

### 4.1. Study Population and Design

A random population sample was examined at baseline in 2003–05 (n = 9360, age 45–69) and re-examined in 2006-08 and 2015-18 in Novosibirsk within the Health, Alcohol and Psychosocial Factors in Eastern Europe study (HAPIEE, http://www.ucl.ac.uk/easteurope/hapiee-cohort.htm, access on 15 October 2023) [55]. The established cohort was followed up until 31 December 2019 for an average of 15.9 years (SD 0.74, median 15.9; IQR 15.18–16.62) for cardiovascular diseases (CVD), cancer, diabetes mellitus, and cause-specific mortality.

The current analysis focused on colorectal cancer (CRC) [ICD-10: C18–C20]. Fatal and non-fatal CRC cases in the cohort were ascertained using the Cancer Register of Novosibirsk city. In addition, multiple sources were used to collect information on all-cause and cause-specific mortality, including the Bureau of Population Registration (ZAGS), the State Statistical Bureau of the Novosibirsk Region, and data obtained during repeated examinations of the cohort (from the address bureau and proxy information on deceased study participants).

### 4.2. Study Sample Selection

This study was designed as a nested case–control study. In a cohort of 9360 persons, during a 16-year follow-up period, 160 new events of colorectal cancer were registered (including repeated events in several subjects). Among them, the set of incident CRC cases comprised 154 cases; from this entire set of incident colorectal cancer cases identified among participants who were cancer-free at baseline and had available DNA samples, we randomly selected a subset of 35 cases for DNA methylation analysis, stratifying by age and sex.

The universal control group for this study included those free from any baseline cancer and CVD who were without the outcome of interest and alive by the census date (31 December 2019). These exclusion criteria for controls were applied to generate a universal control group suitable for several outcomes. Case and control subsets were age- and sex-frequency matched. Finally, after the exclusion of technically inadequate DNA samples or inappropriate DNAm profiling, a CRC group of 35 and a control group of 354 were selected for this study. The general characteristics of the studied groups are shown in Table 1. We also considered an ‘extended control group’, which includes extra 35 participants with incident cancer of a type other than CRC (n = 389) for additional validation and sensitivity analyses.

This study was conducted in accordance with the relevant ethical guidelines and regulations. All study respondents signed informed consent forms for participation; this study protocols were approved by the Ethical Committee of the Research Institute of Internal and Preventive Medicine—Branch of Federal State Budgeted Research Institution, “Federal Research Center, Institute of Cytology and Genetics, Siberian Branch of the Russian Academy of Sciences” (IIPM—Branch of IC and G SB RAS), Protocol № 1 from 14 March 2002 and Protocol № 12 from 8 December 2020.

### 4.3. Data Collection

The baseline data were collected within the HAPIEE project in 2003–2005. The baseline examination was conducted using a standardized interview, with a physical examination and the collection of blood samples. This study included a general assessment of health, including a medical history for hypertension, diabetes mellitus, CVD and other chronic diseases, behavioural and socio-economic factors, and the measurement of blood pressure (BP), anthropometry, and physical performance. The details of the protocol are reported elsewhere [55].

Smoking status was classified as current smoker (at least one cigarette a day), former smoker, and never smoked. Alcohol consumption was categorised into 5 categories by frequency of intake (not-drinking, less than 1 occasion/month, 1–3 occasions/month, 1–4 occasions/week, 5+ occasions/week). Education level was classified into 4 categories (high, secondary, vocational, and primary/less than primary); marital status was categorised into 2 categories (married/cohabiting vs. single/divorced/widowed).

We measured blood pressure (BP) in a sitting position on the right arm three times (Omron M-5) after a 5-min rest, with measurements taken 2 min apart. In this study, we used the average of three BP measurements. Weight, height, and waist and hip circumference were measured with accuracies of 100 g and 1 mm, respectively, and were used to calculate body mass index (BMI, kg/m^2^) and waist-hip ratio (WHR, units).

Blood samples were drawn in a fasting state (minimum 8 h) using the vacuette collection system. After centrifugation, serum was stored in a deep-freezer (minus 80 °C). The concentrations of total cholesterol (TC), triglycerides (TG), high-density lipoprotein cholesterol (HDLC) and glucose in blood serum were measured during one month after sample collection using a KoneLab 300i autoanalyser (Thermo Fisher Scientific Inc., Waltham, MA, USA) by an enzymatic method using the relevant kits. We calculated low-density lipoprotein cholesterol (LDLC) concentration using the Friedewald formula, and we converted the serum glucose concentration to fasting plasma glucose (FPG) value using the formula from EASD, 2007 [56].

Genomic DNA was extracted from whole blood cells by a phenol–chloroform technique [57] and stored in a deep-freezer (minus 70 °C).

### 4.4. DNAm Data Profiling

Whole blood DNAm profiling was performed using Illumina Infinium Methylation EPIC BeadChip arrays following the manufacturer’s recommended protocol (Illumina Inc., San Diego, CA, USA). The arrays were scanned using the iScan Microarray Scanner with an autoloader (Illumina Inc., San Diego, CA, USA) to produce raw signal intensity files (.idat files) in accordance with standard operating procedures.

### 4.5. DNAm Data Preprocessing and Quality Control (QC)

Data was processed using R (v. 4.1.0) [58] and specialized R libraries minfi [59], ChAMP [60] and ENmix [61], following the steps described in [62]. Sample level QC included checking the percentage of low-quality probes (CpGs) per sample based on the signal detection *p*-values and bead count numbers, matching the reported sex with one inferred from DNAm data, and checking that different people were different genetically based on available signals from the highly variable SNP probes. At a probe level, the data was pre-processed using an ssNoob [63] normalisation method implemented in a minfi library. In our analysis, we only included data from samples with less than 1% of low-quality CpGs (i.e., CpGs with detection *p*-values above the threshold 0.01 and bead count numbers below 3), and CpGs that demonstrated good quality (detection *p* < 0.01 across at least 99% samples).

### 4.6. Epigenetic Age and Epigenetic Age Acceleration

Baseline epigenetic age (EA) was calculated using Horvath [25], Hannum [24], PhenoAge [26], Skin and Blood [27], BLUP [29], and Elastic Net (EN) [29] DNAm clocks, implemented in the methylclock R library [64].

Corresponding epigenetic age acceleration (EAA) was calculated as the residuals from regressing epigenetic age (EA) on chronological age (CA) for six measures.

### 4.7. Statistical Analysis

Statistical analysis was conducted using SPSS (v19.0) and R (v4.1.0) software packages. The core dataset consists of 35 CRC cases and 354 controls.

First, in descriptive analysis, we compared CA, EA, EAA, and other baseline characteristics between case and control groups by ANOVA (for continuous variables) and cross-tabulation (for categorical variables).

Second, we used logistic regression to assess odds ratios for incident CRC per decile increase of EAA as continuous variables for six markers. The dependent variable incident CRC. Model 1 was adjusted for baseline age and sex; Model 2 was adjusted for age, sex, and smoking; Model 3 was adjusted for age, sex, smoking, systolic blood pressure (SBP), total cholesterol (TC), BMI, and education level; and Model 4 included the same covariates as Model 3 but with HDLC instead of TC, and this model was additionally adjusted for WHR and fasting plasma glucose (FPG). In addition, we estimated odds ratios for CRC rik by tertiles of EAA values for six markers using the bottom tertile as a reference in logistic regression analyses applying the same covariates for all 4 models.

Also, we conducted several sensitivity analyses. We repeated analyses stratified by sex using the same Models 1–4. To avoid potential reverse effects of the underlined diseases on the reduction of EAA measures, we excluded from analysis CRC cases that occurred within the first 3 years after baseline and repeated logistic regression analyses using the same covariates and models. Finally, we conducted a secondary analysis using the extended control dataset, which includes subjects with incident cancer other than CRC (total sample size no. = 424).

## 5. Conclusions

In this case–control study, we found an association between accelerated baseline epigenetic age by Horvath, Hannum, PhenoAge, and BLUP clocks and the risk of incident colorectal cancer in a West Siberian (Caucasoid) population cohort. These associations were independent of age, sex, and a variety of conventional risk factors for CRC.

Further investigations in larger cohorts of different geographic and ethnic backgrounds, including a diversity of CRC outcomes, are topical, which will help to provide deeper understanding of the relationship between epigenetic age and cancer, and which may have the potential for practical implication in perspective.

## Figures and Tables

**Table 1:** Baseline characteristics of incident colorectal cancer cases and controls from the Russian cohort of the HAPIEE Study (men and women, baseline survey 2003–2005, 45–69 years).

Parameters	Cases (Incident CRC)	Controls	*p*-Value
Observed, n	35	354	
Baseline age, years (mean, SD)	60.2 (7.31)	58.0 (7.05)	*0.088*
Women, no. (%)	18 (51.4)	203 (57.3)	*0.500*
Systolic blood pressure, mmHg (mean, SD)	142.6 (24.78)	141.8 (25.82)	*0.851*
Diastolic blood pressure, mmHg (mean, SD)	90.1 (14.01)	88.6 (13.70)	*0.543*
Body mass index, kg/sqm (mean, SD)	27.7 (4.52)	28.2 (5.21)	*0.578*
Waist/hip ratio, unit (mean, SD)	0.89 (0.09)	0.88 (0.08)	*0.434*
Total cholesterol, mmol/L (mean, SD)	6.32 (1.16)	6.49 (1.28)	*0.445*
LDL cholesterol, mmol/L (mean, SD)	3.95 (1.00)	4.22 (1.13)	*0.167*
HDL cholesterol, mmol/L (mean, SD)	1.73 (0.50)	1.55 (0.33)	*0.005*
TG, mmol/L (mean, SD)	1.42 (0.71)	1.58 (0.81)	*0.244*
Glucose, plasma, mmol/L (mean, SD)	5.98 (1.40)	5.98 (1.47)	*0.988*
Hypertension (%)	22 (62.9)	208 (58.8)	*0.638*
HT treatment (among HT), no. (%)	11 (50.0)	91 (43.8)	*0.575*
Diabetes mellitus type 2, no. (%)	3 (9.1)	34 (9.9)	*0.884*
DMT2 treatment (among DMT2), no. (%)	1 (33.3)	11 (32.4)	*0.972*
Smoking, no. (%)			*0.404*
Present smoker	5 (14.3)	86 (24.3)	
Former smoking	5 (14.3)	48 (13.6)	
Never smoking	25 (71.4)	220 (62.4)	
Frequency of drinking (%)			*0.869*
5+/week	5 (14.3)	47 (13.3)	
1–4/week	12 (34.3)	155 (43.8)	
1–3/month	8 (22.9)	68 (19.2)	
<1/month	9 (25.7)	77 (21.8)	
Non-drinkers	1 (2.9)	7 (2.0)	
Education (%)			*0.815*
Primary	4 (11.4)	33 (9.3)	
Vocational	7 (20.0)	85 (24.0)	
Middle	16 (45.7)	139 (39.3)	
High	8 (22.9)	97 (27.4)	
Marital status (%)			*0.664*
Single	11 (31.4)	99 (28.0)	
Married	24 (68.6)	255 (72.0)	
Epigenetic Age, years, mean (SD)			
Horvath	58.9 (7.82)	53.0 (6.51)	*<0.001*
Hannum	40.3 (6.19)	36.3 (6.41)	*0.001*
PhenoAge	53.3 (8.29)	49.0 (8.02)	*0.003*
Skin and Blood	58.6 (7.29)	58.1 (6.47)	*0.650*
Zhang BLUP	65.8 (7.06)	62.1 (6.39)	*0.001*
Zhang EN	59.6 (7.43)	58.4 (7.13)	*0.347*
Epigenetic Age Acceleration, years, mean (SD)			
Horvath	3.91 (5.45)	−0.43 (4.53)	*<0.001*
Hannum	2.15 (3.41)	−0.24 (4.10)	*<0.001*
PhenoAge	2.29 (4.73)	−0.22 (5.69)	*0.005*
Skin and Blood	−1.03 (3.45)	0.08 (3.73)	*0.091*
Zhang BLUP	1.83 (2.41)	−0.11 (2.90)	*<0.001*
Zhang EN	−0.68 (2.58)	0.08 (3.27)	*0.185*

Presented as mean (SD) or no. (%); SD—standard deviation; *p*-value—from F-Fisher ANOVA or Pearson Chi-square test. LDL cholesterol—low-density lipoprotein cholesterol, HDL cholesterol—high-density lipoprotein cholesterol, TG—triglycerides, HT—hypertension, DMT2—Diabetes mellitus type 2.

**Table 2:** Relationships between incident CRC and epigenetic age acceleration per decile increment of the regression residuals of baseline EA on CA (cases, no. = 35 and controls, no. = 354).

EAA Measure	No., Case/Control	Model 1	Model 2	Model 3	Model 4
		OR (95% CI)	OR (95% CI)	OR (95% CI)	OR (95% CI)
Horvath, per decile	35/354	1.43 (1.22–1.67)	1.43 (1.22–1.68)	1.44 (1.23—1.69)	1.44 (1.21–1.76)
*p-value for trend*		*<0.001*	*<0.001*	*<0.001*	*<0.001*
Hannum, per decile	35/354	1.29 (1.11–1.48)	1.29 (1.11–1.48)	1.29 (1.11–1.49)	1.23 (1.06–1.42)
*p-value for trend*		*0.001*	*0.001*	*0.001*	*0.007*
PhenoAge, per decile	35/354	1.20 (1.05–1.37)	1.23 (1.07–1.41)	1.25 (1.08–1.44)	1.20 (1.04–1.39)
*p-value for trend*		*0.009*	*0.004*	*0.003*	*0.014*
SkinBlood, per decile	35/354	0.88 (0.77–0.99)	0.88 (0.77–0.99)	0.87 (0.76–0.99)	0.86 (0.76–0.99)
*p-value for trend*		*0.044*	*0.040*	*0.029*	*0.033*
BLUP, per decile	35/354	1.34 (1.16–1.55)	1.33 (1.15–1.54)	1.32 (1.14–1.54)	1.35 (1.16–1.57)
*p-value for trend*		*<0.001*	*<0.001*	*<0.001*	*<0.001*
EN, per decile	35/354	0.90 (0.78–1.02)	0.90 (0.79–1.02)	0.89 (0.78–1.01)	0.87 (0.76–0.99)
*p-value for trend*		*0.104*	*0.091*	*0.078*	*0.040*

EAA measures—regression residuals of EA on CA by Horvath; Hanuman, PhenoAge, Skin and Blood, BLUP and Elastic Net clocks; OR—odds ratio; CI—confidence interval; Model 1—adjusted for age and sex; Model 2—adjusted for age, sex, and smoking; Model 3—adjusted for age, sex, smoking, SBP, TC, BMI, and education; Model 4—adjusted for age, sex, smoking, SBP, HDLC, BMI, WHR, FPG, and education.

**Table 3:** Relationships between incident CRC and epigenetic age acceleration by tertiles of the regression residuals of baseline EA on CA (cases, no. = 35 and controls, no. = 354).

EAA Measure	No., Case/Control	Tertiles	Absolute Difference T1–T2 T2–T3	Model 1	Model 2	Model 3	Model 4
				OR (95% CI)	OR (95% CI)	OR (95% CI)	OR (95% CI)
Horvath	35/354	T1(ref)		1.0	1.0	1.0	1.0
		T2	3.96	1.60 (0.37–6.99)	2.12 (0.52–8.71)	2.15 (0.52—8.88)	3.73 (0.72–19.38)
		T3	18.88	12.04 (3.21–45.07)	10.85 (3.15–37.39)	11.38 (3.28–39.56)	16.09 (3.59–72.12)
*p-value for trend*				*<0.001*	*<0.001*	*<0.001*	*<0.001*
Hannum	35/354	T1(ref)		1.0	1.0	1.0	1.0
		T2	3.24	5.95 (1.29–27.47)	6.39 (1.38–29.62)	6.33 (1.36–29.55)	5.35 (1.13–25.26)
		T3	18.07	12.73 (2.90–55.95)	13.57 (3.07–60.00)	13.52 (3.04–60.11)	9.79 (2.15–44.48)
*p-value for trend*				*<0.001*	*<0.001*	*<0.001*	*0.001*
PhenoAge	35/354	T1(ref)		1.0	1.0	1.0	1.0
		T2	4.46	6.59 (1.87–23.22)	6.96 (1.97–24.58)	6.97 (1.97–24.68)	6.66 (1.84–24.06)
		T3	23.79	5.42 (1.50–19.61)	6.20 (1.71–22.52)	6.65 (1.79–24.65)	5.12 (1.35–19.45)
*p-value for trend*				*0.015*	*0.007*	*0.005*	*0.022*
SkinBlood	35/354	T1(ref)		1.0	1.0	1.0	1.0
		T2	2.87	0.57 (0.25–1.35)	0.58 (0.24–1.36)	0.55 (0.23–1.32)	0.55 (0.21–1.40)
		T3	11.98	0.46 (0.19–1.12)	0.45 (0.18–1.09)	0.41 (0.16–1.00)	0.42 (0.16–1.07)
*p-value for trend*				*0.081*	*0.071*	*0.048*	*0.067*
BLUP	35/354	T1(ref)		1.0	1.0	1.0	1.0
		T2	2.29	1.41 (0.43–4.57)	1.44 (0.44–4.68)	1.43 (0.44–4.69)	1.64 (0.48–5.63)
		T3	13.95	5.25 (1.92–14.34)	5.09 (1.86–13.92)	5.27 (1.91–14.52)	5.79 (1.97–17.03)
*p-value for trend*				*<0.001*	*<0.001*	*<0.001*	*<0.001*
EN	35/354	T1(ref)		1.0	1.0	1.0	1.0
		T2	2.54	0.83 (0.37–1.82)	0.76 (0.34–1.70)	0.69 (0.30–1.60)	0.63 (0.26–1.53)
		T3	16.49	0.40 (0.16–1.04)	0.38 (0.15–0.98)	0.36 (0.14–0.94)	0.28 (0.10–0.79)
*p-value for trend*				*0.062*	*0.046*	*0.035*	*0.015*

EAA measures—regression residuals of EA on CA from Horvath; Hanuman, PhenoAge, Skin and Blood; BLUP, and Elastic Net clocks; OR—odds ratio; CI—confidence interval; Model 1—adjusted for age and sex; Model 2—adjusted for age, sex and smoking; Model 3—adjusted for age, sex, smoking, SBP, TC, BMI and education; Model 4—adjusted for age, sex, smoking, SBP, HDLC, BMI, WHR, FPG, and education.

## Data Availability

The data presented in this study are available in tabulated form on request. The data are not publicly available due to the ethical restrictions and project regulations.

## Supplementary Materials

The following supporting information is available online at: https://www.mdpi.com/article/10.3390/ijms25094850/s1.
