# Supplementary material for: Blood-Based Epigenetic Age Acceleration and Incident Colorectal Cancer Risk: Findings from a Population-Based Case–Control Study
Source: Int J Mol Sci. 2024 Apr 29;25(9):4850. doi: 10.3390/ijms25094850 (PMC11084311; doi:10.3390/ijms25094850)
Supplement: Supplementary file 1 [file ijms-25-04850-s001.zip › ijms-2972471-supplementary.pdf]

Supplementary Materials

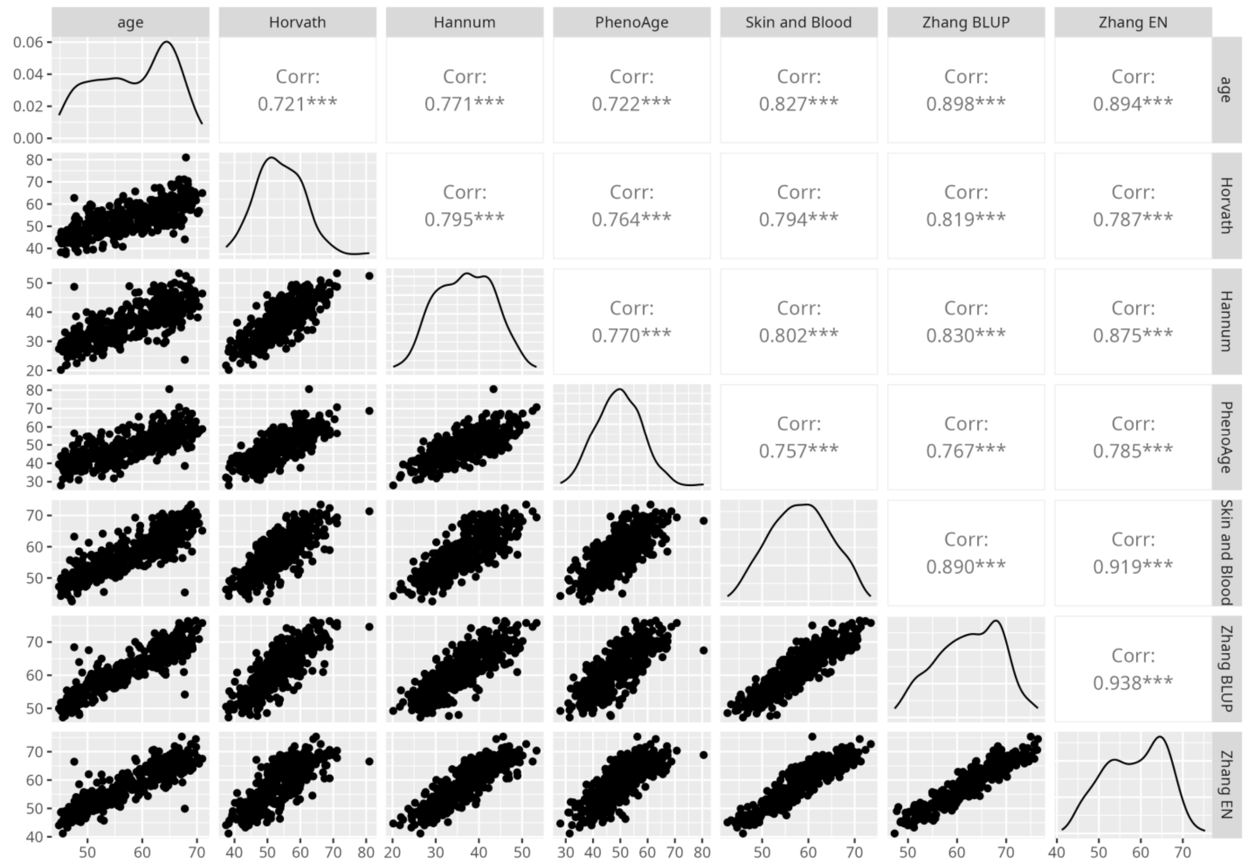

Supplementary Figure S1: Scatterplot matrix visualising the relationships between chronological age and six epigenetic age measures (Horvath, Hannum, PhenoAge, Skin and Blood, Zhang BLUP, and Zhang EN). The lower triangle displays scatterplots for each pair of variables, allowing for the identification of linear or non-linear patterns. The diagonal panels show the density distribution of each variable. The upper triangle reports the Pearson correlation coefficients, quantifying the strength and direction of the linear relationships. The scatterplot matrix confirms that the epigenetic clock outcomes positively correlate with chronological age, with varying strengths of association. It also reveals the inter-relationships among the different epigenetic age measures, highlighting their similarities and differences. Notation: *** p<0.001, ** p<0.01, * p<0.05 (from correlation test).

Supplementary Table S1. Relationship between incident CRC and epigenetic age acceleration, per 1 decile increment of the regression residuals of baseline EA on CA stratified by sex (cases, n =35 and controls, n = 354)

EAA measure	n, case / control	Model 1	Model 2	Model 3
		OR (95%CI)	OR (95%CI)	OR (95%CI)
**Men**				
Horvath, per 1decile	17/151	1.25 (1.02-1.53)	1.24 (1.00-1.53)	1.27 (0.99-1.60)
p value for trend		*0.035*	*0.043*	*0.082*
Hannum, per 1decile	17/151	1.23 (1.00-1.50)	1.23 (1.00-1.50)	1.22 (0.98-1.53)

p-value for trend		*0.047*	*0.046*	*0.082*
PhenoAge, per 1decile	17/151	1.16 (0.96-1.41)	1.24 (1.01-1.52)	1.26 (0.99-1.59)
p-value for trend		*0.132*	*0.039*	*0.051*
SkinBlood, per 1decile	17/151	0.87 (0.72-1.03)	0.86 (0.72-1.03)	0.77 (0.62-0.96)
p-value for trend		*0.111*	*0.104*	*0.019*
BLUP, per 1decile	17/151	1.28 (1.04-1.56)	1.25 (1.02-1.53)	1.28 (1.03-1.60)
p-value for trend		*0.017*	*0.029*	*0.029*
EN, per 1decile	17/151	0.83 (0.70-1.00)	0.82 (0.68-0.99)	0.72 (0.56-0.93)
p-value for trend		*0.052*	*0.044*	*0.012*
**Women**				
Horvath, per 1decile	18/203	1.66 (1.29-2.14)	1.66 (1.29-2.14)	1.80 (1.30-2.48)
p value for trend		*<0.001*	*<0.001*	*<0.001*
Hannum, per 1decile	18/203	1.34 (1.10-1.63)	1.34 (1.09-1.63)	1.23 (1.00-1.50)
p-value for trend		*0.004*	*0.004*	*0.053*
PhenoAge, per 1decile	18/203	1.23 (1.02-1.49)	1.23 (1.03-1.49)	1.15 (0.95-1.41)
p-value for trend		*0.028*	*0.027*	*0.164*
SkinBlood, per 1decile	18/203	0.89 (0.75-1.07)	0.89 (0.74-1.06)	0.90 (0.74-1.09)
p-value for trend		*0.205*	*0.198*	*0.295*
BLUP, per 1decile	18/203	1.41 (1.14-1.74)	1.42 (1.14-1.75)	1.41 (1.13-1.77)
p-value for trend		*0.001*	*0.001*	*0.003*
EN, per 1decile	18/203	0.97 (0.82-1.15)	0.97 (0.82-1.14)	0.92 (0.77-1.11)
p-value for trend		*0.711*	*0.691*	*0.400*

EAA measures - regression residuals of EA on CA by Horvath; Hanuman, PhenoAge, Skin and Blood; BLUP and Elastic Net, correspondently; OR – odds ratio; CI – confidence interval; Model 1: adjusted for age; Model 2: adjusted for age, sex and smoking; Model 3: adjusted for age, sex, smoking, SBP, HDL-C, BMI, WHR, FPG and education;

Supplementary Table S2. Relationship between incident CRC and epigenetic age acceleration, per 1 decile increment of the regression residuals of baseline EA on CA excluding cases occurred during 3 years after examination (cases, n =30 and controls, n = 354)

EAA measure	n, case / control	Model 1	Model 2	Model 3	Model 4
		OR (95%CI)	OR (95%CI)	OR (95%CI)	OR (95%CI)
Horvath, per 1decile	30/354	1.42 (1.20-1.68)	1.42 (1.20-1.68)	1.43 (1.21—1.69)	1.43 (1.19-1.71)
p value for trend		*<0.001*	*<0.001*	*<0.001*	*<0.001*
Hannum, per 1decile	30/354	1.30 (1.11-1.52)	1.30 (1.11-1.52)	1.30 (1.11-1.52)	1.24 (1.05-1.45)
p-value for trend		*0.001*	*0.001*	*0.001*	*0.009*
PhenoAge, per 1decile	30/354	1.23 (1.06-1.43)	1.24 (1.08-1.46)	1.28 (1.10-1.50)	1.24 (1.05-1.45)

p-value for trend		*0.006*	*0.003*	*0.002*	*0.009*
SkinBlood, per 1decile	30/354	0.89 (0.78-1.01)	0.88 (0.77-1.01)	0.88 (0.77-1.00)	0.87 (0.76-1.01)
p-value for trend		*0.078*	*0.072*	*0.058*	*0.065*
BLUP, per 1decile	30/354	1.29 (1.11-1.49)	1.28 (1.10-1.48)	1.26 (1.08-1.47)	1.29 (1.11-1.50)
p-value for trend		*0.001*	*0.001*	*0.004*	*0.001*
EN, per 1decile	30/354	0.93 (0.82-1.07)	0.93 (0.82-1.06)	0.92 (0.81-1.06)	0.90 (0.78-1.04)
p-value for trend		*0.308*	*0.283*	*0.245*	*0.142*

EAA measures - regression residuals of EA on CA by Horvath; Hanuman, PhenoAge, Skin and Blood; BLUP and Elastic Net, correspondently; OR – odds ratio; CI – confidence interval; Model 1: adjusted for age and sex; Model 2: adjusted for age, sex and smoking; Model 3: adjusted for age, sex, smoking, SBP, TC, BMI and education; Model 4: adjusted for age, sex, smoking, SBP, HDL-C, BMI, WHR, FPG and education

Supplementary Table S3. Relationship between incident CRC and epigenetic age acceleration, per 1 decile increment of the regression residuals of baseline EA on CA in extended control, n=424 (cases, n =35 and controls, n = 389).

EAA measure	n, case / control	Model 1	Model 2	Model 3	Model 4
		OR (95%CI)	OR (95%CI)	OR (95%CI)	OR (95%CI)
Horvath, per 1decile	35/389	1.38 (1.19-1.59)	1.37 (1.18—1.58)	1.38 (1.19-1.60)	1.36 (1.17-1.57)
p value for trend		*<0.001*	*<0.001*	*<0.001*	*<0.001*
Hannum, per 1decile	35/389	1.29 (1.12-1.49)	1.29 (1.12-1.49)	1.29 (1.12-1.50)	1.27 (1.09-1.47)
p-value for trend		*0.001*	*0.001*	*0.001*	*0.002*
PhenoAge, per 1decile	35/389	1.21 (1.05-1.38)	1.24 (1.08-1.42)	1.25 (1.08-1.44)	1.24 (1.07-1.43)
p-value for trend		*0.006*	*0.002*	*<0.001*	*0.003*
SkinBlood, per 1decile	35/389	0.88 (0.78-0.99)	0.87 (0.77-0.99)	0.86 (0.76-0.98)	0.86 (0.76-0.98)
p-value for trend		*0.045*	*0.037*	*0.025*	*0.024*
BLUP, per 1decile	35/389	1.36 (1.17-1.57)	1.35 (1.16-1.56)	1.35 (1.16-1.57)	1.36 (1.17-1.58)
p-value for trend		*<0.001*	*<0.001*	*<0.001*	*<0.001*
EN, per 1decile	35/389	0.91 (0.80-1.03)	0.90 (0.80-1.02)	0.89 (0.79-1.02)	0.87 (0.78-1.02)
p-value for trend		*0.126*	*0.105*	*0.084*	*0.087*

EAA measures - regression residuals of EA on CA by Horvath; Hanuman, PhenoAge, Skin and Blood; BLUP and Elastic Net, correspondently; OR – odds ratio; CI – confidence interval; Model 1: adjusted for age and sex; Model 2: adjusted for age, sex and smoking; Model 3: adjusted for age, sex, smoking, SBP, TC, BMI and education; Model 4: adjusted for age, sex, smoking, SBP, HDL-C, BMI, WHR, FPG and education;
